# Supplementary material for: The Interactive Effect of Genetic and Epigenetic Variations in FKBP5 and ApoE Genes on Anxiety and Brain EEG Parameters
Source: Genes (Basel). 2022 Jan 18;13(2):164. doi: 10.3390/genes13020164 (PMC8872390; doi:10.3390/genes13020164)
Supplement: Supplementary file 1 [file genes-13-00164-s001.zip › genes-1528831-supplementary.pdf]

Primers for pyrosequencing promotor region FKBP5 (region, hg19 6:35,656,792-35,656,628)

FKBP5-prom-F BIOTIN-5'ATAGTTTYGGGGTTTTATGGAAGG3'

FKBP5-prom-R 5'TACTCCRCTAACCCTTCAACCC3'

Table S1. Allele frequency differences for rs1360780 in FKBP5 gene

Groups	Allele freq. differences (chi square, p-value)
**ND vs. Nona+**	chi2=0.03 p=0.85360 (P)
**ND vs. Patients with AD**	chi2=0.59 p=0.44236(P)
**Nona+ vs. Patients with AD**	chi2=0.46 p=0.49633 (P)

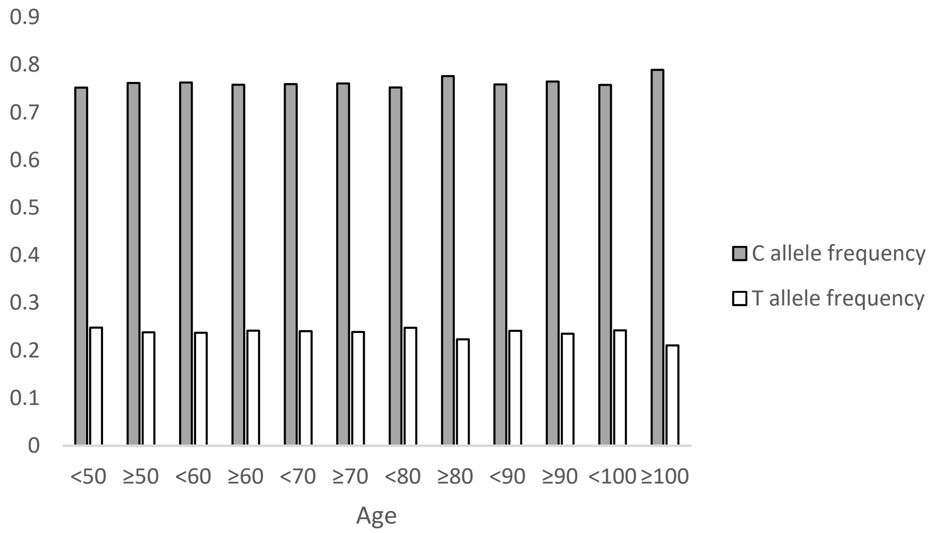

Figure S1. Allele frequency differences for rs1360780 in *FKBP5* gene in ND and Nona+ groups depending on the age

Table S2. Allele frequency differences for rs1360780 in FKBP5 gene in ND and Nona+ groups depending on the age

Groups	Allele freq. differences
**19-39 vs. 40-59 years**	chi2=0.46 p=0.49619 (P)
**19-39 vs. 60-79 years**	chi2=0.00 p=0.99463 (P)
**19-39 vs. 80-107 years**	chi2=0.63 p=0.42787 (P)
**40-59 vs. 60-79 years**	chi2=0.80 p=0.36979 (P)

**40-59 vs. 80-107 years**	chi2=0.01 p=0.92773 (P)
**60-79 vs. 80-107 years**	chi2=1.20 p=0.27417 (P)

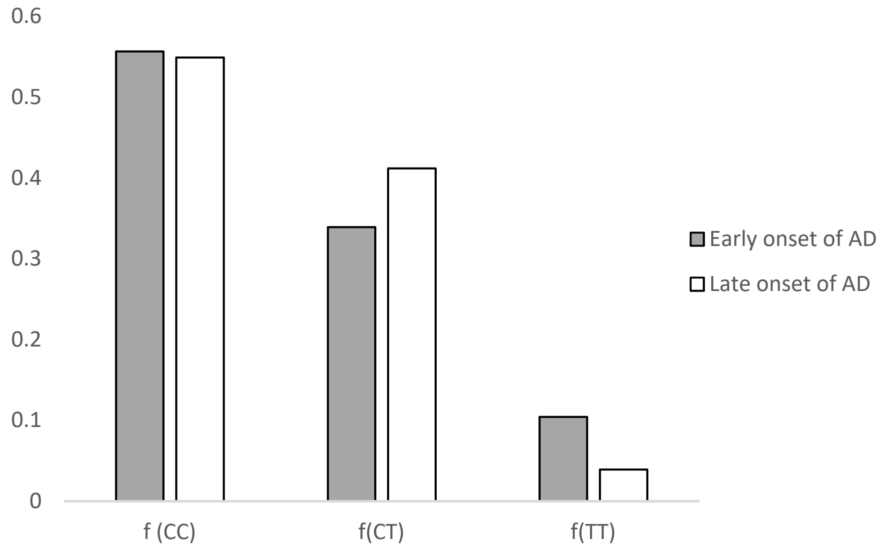

Figure S2. Frequencies of CC, CT, and TT genotypes in rs1360780 FKBP5 in patients with early (< 65) and late (≥ 65) forms of AD

Table S3. Allele frequencies of ε2/ε3/ε4 polymorphism in APOE gene

Group	Number	Genotypes frequency						Allele frequency			HWE, p-value (Pearson)
		ε2/ε2	ε2/ε3	ε2/ε4	ε3/ε3	ε3/ε4	ε4/ε4	ε2	ε3	ε4	
**ND**	479	0	0,150	0,013	0,660	0,165	0,012	0,081	0,817	0,101	0,584441
**Nona+**	100	0	0,180	0,020	0,630	0,150	0,020	0,100	0,795	0,105	0,339557
**Patients with Alzheimer's disease**	221	0	0,063	0,009	0,394	0,407	0,127	0,036	0,629	0,335	0,330537

Table S4 Allele frequency differences for ε2/ε3/ε4 polymorphism in *APOE* gene

Groups	Allele freq. differences (chi square, p-value)
**ND vs . Long-livers**	chi2=0.03 p=0.87340 (P)
**ND vs. Patients with AD**	chi2=114.31 p=1.116e-26 (P)
**Long-livers vs. Patients with AD**	chi2=37.51 p=9.115e-10 (P)

Table S5. Demographic characteristics of participants with EEG

	All participants		Men		Women	
	FKBP CC	*FKBP CT&TT*	*FKBP CC*	*FKBP CT&TT*	*FKBP CC*	*FKBP CT&TT*
N	94	67	31	29	63	38
Age, years	49.8±1.8	47.4±2.0	47.2±3.4	45.8±3.3	51.1±2.0	48.6±2.4

The data are presented as the means and standard errors

Table S6. Log-transformed relative power (mean and SE) of EEG bands in the non-demented men and women with the *FKBP5 CC* and *FKBP5 CT&TT* genotypes.

Effect	Level of Factor	Level of Factor	N	Delta Mean	Delta SE	Theta Mean	Theta SE	Alpha Mean	Alpha SE	Beta1 Mean	Beta1 SE	Beta2 Mean	Beta2 SE
Total			161	-2,18	0,05	-1,41	0,03	-0,23	0,05	-1,90	0,03	-2,49	0,05
Sex	Men		60	-2,05	0,08	-1,36	0,05	-0,34	0,08	-1,89	0,05	-2,44	0,08
Sex	Women		101	-2,26	0,05	-1,44	0,04	-0,17	0,06	-1,90	0,05	-2,52	0,06
FKBP5	*CC*		94	-2,21	0,06	-1,44	0,04	-0,20	0,06	-1,91	0,05	-2,53	0,06
FKBP5	*CT&TT*		67	-2,14	0,07	-1,36	0,05	-0,28	0,08	-1,89	0,05	-2,43	0,08
Sex * *FKBP5*	Men	*CC*	31	-2,18	0,10	-1,40	0,08	-0,22	0,09	-1,95	0,08	-2,60	0,12
Sex * *FKBP5*	Men	*CT&TT*	29	-1,90	0,12	-1,32	0,07	-0,48	0,12	-1,83	0,07	-2,27	0,11
Sex * *FKBP5*	Women	CC	63	-2,22	0,08	-1,46	0,05	-0,19	0,08	-1,88	0,06	-2,49	0,08
Sex * *FKBP5*	Women	*CT&TT*	38	-2,32	0,07	-1,39	0,08	-0,12	0,11	-1,94	0,07	-2,55	0,11
